# Macro-economic determinants of the IPO waves: An assessment of G-7 countries

**DOI:** 10.12688/f1000research.122849.2

**Published:** 2024-01-29

**Authors:** MUSTAFA ÖZYEŞİL, Esin Benhür Aktürk

**Affiliations:** 1 Business Administration, Istanbul Aydin Universty, Istanbul, Turkey; 2 Management organization, İstanbul Aydin University, İstanbul, Turkey

**Keywords:** IPO Wave, Stock Returns, Panel Data, Random Effect Model, Driscoll-Kraay Standard Error, New Product Development, Operations Management, Organization Development Change, Continuous Improvement

## Abstract

**Background:**

The objective of this study is to explore the macroeconomic factors considered by companies in scheduling initial public offerings (IPOs).

**Methods:**

The panel data analysis method was employed to investigate the relationship between the frequency of IPOs and selected macroeconomic indicators within the stock markets of G-7 countries spanning from 1999 to 2020. An econometric model utilizing a random effect approach was utilized, employing the Driscoll-Kraay resistant standard estimator to address deviations.

**Results:**

The analysis revealed that stock market returns exert a statistically significant positive influence on the volume of public offerings.

**Conclusions:**

Based on these findings, it can be inferred that companies strategically time their IPOs during periods characterized by relatively high stock market returns.

## Introduction

Equity financing through Initial Public Offerings (IPOs) stands as a strategic financial maneuver within capital markets. The literature debates the pros and cons associated with IPOs for companies. The act of going public endows a company with the status of a publicly traded entity, listing its stocks on the exchange, thereby fostering a liquid secondary market among investors. Furthermore, post-issuance, publicly traded firms gain the advantage of raising capital under more favorable terms in subsequent periods ( Ibbotson and Ritter, 1995). Conversely, public offerings entail numerous direct and indirect costs, encompassing intermediary fees paid to financial institutions and regulatory listing fees, deemed one-time expenditures, alongside ongoing obligations to furnish information to regulatory bodies ( Ibbotson and Ritter, 1995). Considering additional factors such as shared management and ownership during the IPO process, companies must diligently weigh the associated advantages and disadvantages.

The realm of corporate finance places paramount importance on public offerings, evidenced by extensive literature examining various facets of IPOs. Studies have delved into macro- and micro-level determinants precipitating companies’ decisions to go public, as well as the short- and long-term price performance of stocks post-IPO. For instance, researchers like Chemmanur and He (2011), Rydqvist and Högholm (1995), and Draho (2004) have scrutinized rationales behind companies going public and the frequency of such offerings. Others, such as Loughran and Ritter (2004), Booth and Chua (1996), Ljungqvist (2007), Ritter and Welch (2002), and Ritter (1984), have focused on price movements and their determinants. Studies exploring the causes and frequency of public offerings have spawned hypotheses like hot and cold IPOs, and various theories such as the Winner’s Curse proposed by Rock (1986) have emerged concerning post-IPO price performance.

This study centers on examining the macroeconomic determinants influencing the frequency of IPOs, specifically delving into which indicators companies consider when timing their public offerings. Employing panel data analysis, the research spans the period 1999-2020 across G-7 countries.

The volume, count, and returns of IPOs are shaped by firm-specific attributes, the macroeconomic milieu, and intermediary institutional behaviors. Notably, investment banks wield extensive experience in managing public offerings, producing pivotal information guiding market participants during these processes. Particularly during periods of heightened issuance and initial returns, investment banks’ information enhances IPO quality, enabling lower-quality firms to go public and bolstering secondary market prices, synchronizing increased IPO volumes and initial returns ( He, 2007).

The subsequent sections of this study entail a review of international literature on the subject, an analysis of the relationship between the frequency of public offerings and the selected macroeconomic indicators, and a comprehensive interpretation and evaluation of the findings.

### Literature review

The majority of the studies in the literature have analyzed the trend of IPOs in terms of number and volume and have created the concepts known as IPO Wave and Hot and Cold Issue markets in the literature. In most of the studies in the literature, the markets in which the number and volume of IPOs increase gradually are defined as “hot issue markets”, while the markets where the number and volume of IPOs decrease are defined as “cold issue markets”. In hot issue markets, many companies can plan their IPOs for this period, as the investor’s demand for the stock market is increasing. This way, the company makes a successful public offering and the investors have the opportunity to obtain abnormal returns in the short term thanks to the high discounts they obtain.

Pástor and Veronesh (2005) developed an optimal IPO timing model for firms. They emphasized that many studies in the literature explained the change in IPO volume over time with the concept of market inefficiency, where IPOs are made when stocks are overvalued. Contrary to previous literature, they created a model in which the fluctuation in the volume of initial public offerings occurs without any mispricing mechanism and the volume of the IPO is more closely related to recent changes in stock prices rather than the level of market stock prices. In the model, the variables that companies take into account during the public offering are, respectively: expected market return, expected aggregate profitability and prior uncertainty about the average future profitability of IPOs. According to the results of the analysis, they determined that there were high market returns before the IPO waves and low market returns after the IPO waves.

Buttimer, Hyland and Sanders (2005), in their study, comparatively examined the waves of IPOs in the real estate investment trust (REIT) sector and the waves of general initial public offerings. In terms of initial returns, it was observed that the REIT sector provided lower returns than the general market average after the IPO waves. In addition, the long- term underperformance anomaly, which is frequently observed in stock markets according to many studies such as Ritter (1991), Loughran and Ritter (1997), Levis (1993), Schultz (2003) among others, has not been observed in the REIT sector. Based on the findings of their study, authors stated that the Capital Demand Hypothesis best describes the REIT public offering market.

Yung, Colak and Wang (2008) created a model in which real investment opportunities that change over time cause adverse selection in the market for IPOs. As a result of the analysis, it has been determined that economic expansions are highly correlated with the number of companies that go public, which is positively correlated with underpricing. In line with the model they created, greater cross-sectional return variance and higher delisting rates were detected for hot market public offerings.

Christoffersen, Nain and Tang (2010) examined the waves and the quality of IPOs. They stated that the quality of the public offering will not be the same in the first and later periods of the IPO wave. They noted that in the early stages of the IPO wave, the average quality of IPO stock is low when initial returns and IPO demand are low. However, later in the IPO wave, when initial returns and IPO demand are low, IPOs have better operating performance, higher market share, and consequently higher long-term abnormal returns. According to the results of the study, they found that institutional investors benefit from short-term high returns in early public offerings, but they prefer IPOs that are done in later stages of the IPO wave thanks to their higher quality in the long run.

Tran and Jeon (2011) analyzed the macroeconomic factors that were effective on the initial public offerings in the US capital market during the 1970-2005 period. By applying time series econometric methods in the analysis, they determined the long-term balance between macroeconomic variables and public offerings. As in many studies, Tran and Jeon also found that stock market return performance and volatility have the most significant impact on the timing of the IPO. In their study, they also examined the effects of interest rates and bond market returns. They found that there are not only long-term but also short-term statistically significant relationships between macroeconomic variables and IPO activities.

Angelini and Foglia (2018) examined the short- and long-term relationships between initial public offerings and macroeconomic factors in the UK stock market for the period 1996-2016. In the analysis, they sought answers to the questions of how macroeconomic conditions affect the initial public offerings and how long the recent shock effect lasted. Business cycle, volatility, interest rates and stock returns were used as macroeconomic variables. According to the results of the correlation analysis made in the study, it was determined that the business cycle, volatility and interest rate variables could explain the change in the number of IPOs. However, unlike many studies in the literature, no significant effect of stock market performance on the number and timing of IPOs could be detected.

Thanh (2020) used macro econometric models to examine the cycles of initial public offerings in US stock markets. According to the results of the time series analysis they applied in their studies, they determined the strong and negative effect of macroeconomic uncertainty on the public offering activity. It has been determined that a one-unit standard deviation increase in macroeconomic uncertainty reduces the monthly number of initial public offerings by approxi- mately four units in the long run. In the analysis, they also found that both the decrease in the number of IPO applications and the increase in withdrawn IPOs contributed to the decrease in the number of IPOs in response to an uncertainty shock.

Carosi and Mengoli (2021) analyzed local IPO waves in their studies. They analyzed public offerings on the basis of region and sector. They found that the waves of public offerings overlapped on a sectoral and regional basis. In other words, public offerings on a sector basis are similar to public offerings on a regional basis. They observed that IPOs at the beginning of the IPO wave were equally priced lower than IPOs at the end of the wave. According to the results of the analysis, it was determined that the IPO decision is sensitive not only to the high valuations of the companies in the same sector, but also to the high valuations of the companies in the same region but in different sectors.

The research conducted by Cerpentier et al. (2022) focuses on the impact of market timing on the capital structure of private firms raising initial equity crowdfunding (ECF). Unlike prior finance studies that primarily concentrate on publicly traded or IPO firms, this study uniquely delves into the capital structure dynamics of privately held entities. Analyzing firms funded through the two major UK ECF platforms, Crowdcube or Seedrs, the study differentiates between ECF campaigns launched in hot markets, characterized by high ECF volumes, and those in cold markets. Results indicate that firms in hot markets set higher targets, collect more overfunding, and consequently raise more equity capital compared to firms in cold markets. Surprisingly, there are no discernible differences in leverage ratios between firms in hot and cold markets during the year of the ECF campaign, contradicting the expectations derived from market timing theories of capital structure. This unexpected finding is attributed to hot-market ECF firms contemporaneously rebalancing their capital structure by attracting more debt, particularly financial debt.

Mehmood et al. (2021) comprehensively examines the phenomenon of underpricing in Initial Public Offerings (IPOs) across developed, developing, and emerging markets. Through an extensive analysis of existing literature, the review highlights a prevalent trend of higher underpricing in emerging markets compared to developing and developed markets. It identifies this discrepancy as emerging market issuers interpreting underpricing as a signal of quality IPOs, particularly where information asymmetry plays a significant role. Additionally, the review identifies various country-specific factors influencing the scale of underpricing across different markets, providing insight into potential primary determinants for future research.

Cao et al. (2021) investigate how market frictions affect managerial incentives and the organizational structure of new hedge funds. Their study develops a model wherein new managers actively seek accredited investors and exhibit stronger incentives to acquire managerial skills when faced with low investor demand. Empirical analysis using a merged database indicates that cold fund inceptions, identified ex-ante with low investor demand, outperform existing hedge funds, while hot inceptions facing high demand do not perform as well. This sheds light on the competitive advantages of cold stand-alone inceptions over various types of family-affiliated inceptions.

## Methods

### Dataset and sample structure

In the study, we examined whether macroeconomic variables have an effect on the frequency of public offerings. Since the aim is to determine the factors affecting the IPO wave through developed capital markets, the sample consists of G-7 countries. The analysis period covered the period 1999-2020, and annual data were used in the analysis.

Macroeconomic indicators such as annual consumer inflation, stock market returns and economic growth over the years (GDP growth) were used as independent variables. Explanations regarding on variables included in analysis are provided in Table 1 as follows.

**Table 1.** Descriptions of variables.

Variable	Description
Consumer inflation	Inflation is measured through consumer price index. This index indicates annual change in % in cost to average individual of buying goods and services.
Stock market returns	Average annual return over the daily closing prices of each country's national share index.
GDP growth	Annual change in % of GDP based on market prices and constant local currency.

**Source:** https://databank.worldbank.

Our preliminary expectations were a negative relationship between consumer inflation and the frequency of public offerings, and a positive relationship between stock returns and economic growth and frequency of public offerings.

### Methodology

In the study, the relationship between the IPO waves (frequency) and macroeconomic variables were analyzed in terms of country sections. For this reason, panel data analysis method was used. In the analysis, it was deemed appropriate to apply the random effect model as the econometric model. While macroeconomic variables were included in the analysis as independent variables, the number of IPOs was included in the analysis as a dependent variable.

Since there was a cross-section dependency between the series in the analysis, the stationarity of the data was measured with the Peseran (2007) unit root test, which is one of the second-generation unit root tests. Afterwards, the Hausman test was applied to determine the econometric model to be applied. According to the results of the test, the application of the random effect model was deemed appropriate and the Driscoll-Kraay resistant standard estimator developed against deviations was used in the application of the model.

## Results

### Descriptive statistics

Descriptive statistics for the sample used in the study are shown in Table 2 below.

**Table 2.** Descriptive statistics.

Descriptive statistics	IPOs	GDP growth	Stock returns	Inflation
Mean	84.94156	0.012248	0.036149	0.014812
Median	56	0.01695	0.05445	0.016
Maximum	486	0.0687	0.3999	0.039
Minimum	0	-0.094	-0.3632	-0.013
Std. dev.	93.52817	0.02532	0.157667	0.01052
Skewness	1.996234	-1.804778	-0.318524	-0.237892
Kurtosis	7.271059	7.328356	2.391868	2.596243
Jarque-Bera	219.3329	203.8162	4.977113	2.498583
Probability	0	0	0.08303	0.286708
Sum	13081	1.8862	5.5669	2.281
Sum Sq. Dev.	1338370	0.098089	3.803388	0.016934
Observations	154	154	154	154

Correlation analysis is one of the most typical analyzes used to examine the relationship between variables. Pearson’s correlation analysis was used in this study, as the data showed normal distribution.

The correlation relationship between the variables is shown in Table 3 below.

**Table 3.** Correlation analysis.

	Stock-returns	Inflation	GDP-growth	IPOs
Stock-returns	1			
Inflation	0.05	1		
GDP-growth	0.49	0.32	1	
IPOs	0.30	0.12	0.25	1

According to the results of the correlation test, since the degree of correlation between the variables varies between 0.05 and 0.49, there was a positive but weak relationship. The degree of correlation differed according to the variables used. For example, there was a very weak correlation of 0.05 between stock returns and inflation, while a weak correlation of 0.49 between the GDP growth and stock returns. A strong and very strong relationship between the variables in the correlation analysis should have a degree of correlation between 0.70-0.89 and 0.90-1.00, respectively. As a result, according to the results of the analysis, there is a weak relationship between the variables in this sample.

### Unit root test

In order not to encounter the spurious regression problem in the analysis, it is necessary to measure the stationarity of the series before the regression analysis. However, in order to determine which stationarity test will be used, a cross-section dependency test should be applied. According to the result of the cross-sectional dependence, the appropriate unit root test was applied. Cross-section dependence was tested with Pesaran (2004) cross-section dependence method.

The hypotheses of this test were as follows:

*H*0: There is no cross-section dependence between the series.

*H*1: There is a cross-section dependence between the series.

The results of the test are shown in Table 4 below:

**Table 4.** Pesaran (2004) cross-section dependency (CD) test.

Variable	CD-test	p-value	Correlation coefficient	abs (corr)	Result
IPOs	7.84	0.00	0.365	0.366	H0 cannot be accepted
StockReturns	16.49	0.00	0.767	0.767	H0 cannot be accepted
Inflation	10.24	0.00	0.476	0.51	H0 cannot be accepted
GDPGrowth	17.84	0.00	0.83	0.83	H0 cannot be accepted

According to the results of the test, there was a cross-sectional dependence between the series. As a consequence, Pesaran (2007) second generation unit root test was applied, taking this stiuation into account.

The hypotheses of these tests were as follows:

*H*0: Series are not stationary.

*H*1: Series are stationary.

The results of the test are shown in Table 5 below.

**Table 5.** Unit root test results.

Variable	p-value		
	Origin level	First difference	Decision
IPOs	0.636	0.00	I(1)
Stock returns	0.09	-	I(0)
Inflation	0.072	0.00	I(1)
GDP growth	0.04	-	I(0)

According to the unit root test results, IPOs and Inflation were found to be stationary after taking the first difference, while the GDP growth and stock returns variables were found to be stationary at level values. Therefore, non-stationary IPOs and inflation variables were included in the regression analysis after taking their first difference. For this reason, a cointegration test was not be applied in the study.

### Econometric model

A Hausman test was used to determine the econometric model to be established. The Hausman test results are summarized in Table 6 below.

**Table 6.** Hausman test results.

Chi ^2^ (3)	p-value	Decision
4.74	0.8956	Random effect model is used

According to this result, the random effect model was used as the econometric model.

Tests related to autocorrelation and heteroscedasticity problems in the model were performed in Table 7 and Table 8 below.

**Table 7.** Autocorrelation test.

F	p-value	Decision
1.874	0.2201	There is no autocorrelation problem.

**Table 8.** Heteroscedasticity test.

Chi ^2^	p-value	Decision
4.74	0.0295	There is no autocorrelation problem.

In the model, macroeconomic variables were used as independent variables, and IPO numbers were used as dependent variables, and a random effect regression analysis with the Driscoll-Kraay resistant standard estimator was applied.

Regression analysis results are shown in Table 9 as follows.

**Table 9.** Regression with Driscroll - Kraay standard errors. GLS: generalised least squares.

Regression with Driscroll - Kraay standard errors				Number of observations = 154		
Method: Random effects GLS regression				Number of groups = 7		
Group variable: code				Observations per group:		
R-square				minimum = 21		
within = 0.2037				average = 21.0		
between = 0.1687				maximum = 21		
overall = 0.1959				Wald Chi ^2^ (3) = 9.43		
				Probability > Chi ^2^ = 0.0241		
**IPOs**	**Coefficient**	**Robust standard error**	**z**	**p > (z)**	**[95% Confidence interval]**	
GDP growth	-203.6567	225.7047	-0.90	0.367	-646.0297	238.7164
Stock returns	190.9641	78.77502	2.42	0.015	36.56787	345.3603
Inflation	-669.5706	412.4192	-1.62	0.104	-1477.897	138.7561
_constant	-6.434738	3.141505	-2.05	0.041	-12.59197	-.2775006

According to the results of the regression analysis, stock returns is the only variable that has a positive and significant effect on the number of IPOs. The effects of all other variables on the frequency of IPOs were not found significant. Based on the analysis findings, it can be stated that companies take into account the stock returns variable in timing the public offering. The correlation between stock returns variable and IPOs was found to be positive. In other words, as the stock market index returns increase, companies may choose to go public in order to enter the hot public offering market and maximize their issuance proceed (or reduce the underpricing degree of public offerings). The findings are compatible with many studies in the previous literature, especially Pástor and Veronesh (2005), and Tran and Jeon (2011).

## Discussion

The studies in the literature have approached IPO waves from various perspectives, providing a broad outlook. For instance, the work by Pástor and Veronesh (2005) explicates IPO waves in the context of market efficiency and stock valuation, presenting an alternative model. This model highlighted companies’ tendencies to focus more on recent stock prices.

In contrast, this study aimed to correlate IPO waves in G-7 countries with specific macroeconomic factors. The analysis notably demonstrated the positive influence of stock returns on the number of IPOs. However, it found that other macroeconomic variables did not significantly demonstrate such an effect.

While many studies in the literature delve into specific sectors or markets, this study took a more general perspective, examining the relationship between IPO waves in G-7 countries and distinct economic conditions. By focusing on the role of macroeconomic factors in companies’ IPO timing, this research potentially contributes to a better understanding of the timing of public offerings and the impact of macroeconomic conditions on IPO decisions.

## Conclusions

IPOs are one of the most important methods used by companies as long-term equity financing in the capital market. In an IPO, the issuer receives issuance income in exchange for shares sold to investors. Investors, on the other hand, become a shareholder of a newly publicly traded company, often taking advantage of the discounted issue price, and as long as they hold the stock in the future, they will receive dividend income or can get capital gain by selling the discounted shares at a higher price. Considering the above-mentioned advantages and long-term results of public offerings, it was found that this strategic financing process has an important place in the financial success of companies. For this reason, the success of the IPOs is critical for all parties in the IPO process as well as for the general market functioning. Due to the importance of IPOs, there have been numerous studies on IPOs in the literature. The majority of the studies focused on the short- and long-term price performances and determining factors of IPOs, as well as the cycle of IPOs.

In this study, macroeconomic factors that were thought to be effective on the cycle of IPOs were examined, and especially whether economic growth, inflation and stock market returns are determinants on the number of IPOs. The analysis was applied to the G-7 countries representing the developed world economies, for the period 1999-2020.

In the analysis, first of all, the stationarity of the series was tested with the Pesaran (2007) unit root test, which takes into account the cross-section dependence. The series that were not stationary at the values level values were included in the regression analysis by considering their primary difference. Before the regression analysis, we tested whether the problems of autocorrelation and heteroscedasticity existed in the model, and it was determined that there were no such problems in the model. A Hausmann test was applied in the selection of the econometric model to be established, and according to the results obtained, it was deemed appropriate to use the random effect model. Regression analysis was performed using the Driscoll-Kraay resistant standard estimator, which was resistant to deviations and errors.

According to the results of the analysis, it was determined that only the stock returns variable, among the independent variables in the model, had a significant effect on the number of initial public offerings. The stock returns variable has a positive effect on the number of IPOs. In other words, as stock return increases, the number of IPOs also increases. Based on this finding, it can be stated that stock returns is one of the macroeconomic factors that affect companies’ public offering decision. In other words, companies prefer to go to public offerings during periods when stock market returns increase. The inflation and GDP growth variables, which were the other variables investigated in the analysis, could not have a significant effect on the frequency of the initial public offering.

In this analysis, only the macroeconomic determinants, which are thought to be effective on the IPO waves, were examined. In addition, more comprehensive and meaningful results can be obtained by including microeconomic factors that may affect the number of IPOs in the analysis. A study on developing economies in addition to developed world economies will be extremely useful in terms of showing comparatively how macro- and microeconomic factors change according to the level of development of countries.

### Research limitations and further research suggestions

The study was limited to a sample comprising solely G-7 countries, representing developed markets. Consequently, the study lacks the capacity for comparative analysis and interpretation across different country groups, given the absence of results from emerging or underdeveloped markets. To attain more comprehensive and comparative outcomes, it is advised to replicate similar analyses within other country cohorts. Furthermore, the study exclusively incorporated macroeconomic factors believed to impact the volume of IPOs, while disregarding micro-level company-specific factors. Incorporating micro factors within the model stands to yield more meaningful results.

### Implications

This study correlated IPO waves in G-7 countries with specific macroeconomic factors. The analysis revealed that stock returns have a significant positive impact on increasing the number of IPOs. These findings underscore the importance for companies to consider stock market performance in their IPO timing, emphasizing the role it plays in public offerings. Conversely, other macroeconomic variables did not demonstrate a similar effect.

These findings yield a range of significant implications for companies and investors operating in financial markets. Particularly, the necessity for companies to focus on stock returns in their IPO timing is highlighted, enabling a better understanding of factors influencing the performance and competitive advantages of potential IPO companies.

Furthermore, this research may assist in better comprehending the influence of economic conditions and macroeconomic variables on companies’ IPO decisions. This could aid businesses and investors in making strategic decisions based on a more robust foundation in timing their public offerings.

## Data availability

### Underlying data

Inflation and GDP growth rate data were retrieved from WorldBank databank and filtered for the G-7 countries for the period of interest ( https://data.worldbank.org/indicator/FP.CPI.TOTL.ZG). In the menu on the right of the home page, the indicators for sample countries can be selected. No account creation is needed.

IPO numbers were retrieved from Statista. Scholars don’t have to create account and make subscription on Statista when they are downloading IPO numbers for each country seperately. For multiple countries and periods, the system requires to create an account.

Stock returns were retrieved from Investing.com. In thesearch box located at the top of the home page of investing, equity code is written and profile page of related stock wil be opened. The historical data section is located just above the price chart. After determining start and end dates all information regarding on closing price, opening price, highest and lowest will be visible.

All data used are open access.
