# Supplementary material for: Macro-economic determinants of the IPO waves: An assessment of G-7 countries
Source: F1000Res. 2024 Jan 29;11:763. Originally published 2022 Jul 8. [Version 2] doi: 10.12688/f1000research.122849.2 (PMC11791398; doi:10.12688/f1000research.122849.2)
Supplement: Peer review history

# Reviewer report: Reviewer response for version 2

**DOI:** 10.5256/f1000research.161194.r362655

**Published:** 2025-02-03

**Authors:** Kamil Gemra

**Affiliations:** 1 SGH Warsaw School of Economics, Warsaw, Poland

The authors have undertaken an important research topic. The issue of factors influencing the IPO process is widely commented on in the literature. Here, it would be worth citing more new literature, especially that published after 2020. I think that the authors could supplement this.

 The work is interesting because it draws attention to macroeconomic factors. However, their selection is controversial. First of all, the level of interest rates is missing in this study. After all, this mainly affects investors' decisions regarding the asset class in which they invest their capital. Another factor is the exchange rate and there is nothing about it here either. Among the listed macroeconomic factors, attention is often drawn to the level of unemployment. These factors are not in the article. If the authors believe that they are not worth including, they should write why they do not want to include them.

 It follows from the entire work that the only factor is the rate of return on the stock market. I think it is obvious that the number of IPOs will increase during a bull market and decrease during a bear market. Returning to the previous comment, I wonder why the number of macroeconomic factors is small, and the factor related to a given stock market is controversial. Maybe it is worth expanding the number of factors and then approaching the study.

Is the work clearly and accurately presented and does it cite the current literature?

Partly

If applicable, is the statistical analysis and its interpretation appropriate?

Yes

Are all the source data underlying the results available to ensure full reproducibility?

Yes

Is the study design appropriate and is the work technically sound?

Yes

Are the conclusions drawn adequately supported by the results?

Partly

Are sufficient details of methods and analysis provided to allow replication by others?

Yes

Reviewer Expertise:

My research areas are financing, especially with regard to the capital market. Therefore, the subject of IPO is close to me.

I confirm that I have read this submission and believe that I have an appropriate level of expertise to confirm that it is of an acceptable scientific standard, however I have significant reservations, as outlined above.

# Reviewer report: Reviewer response for version 2

**DOI:** 10.5256/f1000research.161194.r241862

**Published:** 2024-12-16

**Authors:** Prof.Dr.Cemal Zehir

**Affiliations:** 1 Yıldız Technical University, Istanbul, Turkey

The article “Macro Economic Determinants of the IPO Waves: An Assessment of G7 Countries” is a highly insightful contribution to the field of financial economics. By examining the macroeconomic factors that drive IPO waves across G7 countries, the authors offer a unique perspective that bridges global economic trends with financial market activity.

 The comprehensive analysis of variables such as GDP growth, interest rates, and market sentiment provides a well-rounded understanding of how broader economic conditions influence the timing and frequency of IPOs. The comparative approach across G7 countries adds significant value, highlighting both commonalities and country-specific dynamics.

 Furthermore, the paper is exceptionally relevant for policymakers, investors, and scholars alike, offering actionable insights into how macroeconomic policies and global economic shifts can impact equity markets. The authors’ rigorous methodology and clear presentation make this work not only academically robust but also practically accessible.

 Overall, this paper is a commendable effort that advances our understanding of IPO waves and their macroeconomic underpinnings, setting the stage for further research in this critical area.

Is the work clearly and accurately presented and does it cite the current literature?

Yes

If applicable, is the statistical analysis and its interpretation appropriate?

Yes

Are all the source data underlying the results available to ensure full reproducibility?

Yes

Is the study design appropriate and is the work technically sound?

Yes

Are the conclusions drawn adequately supported by the results?

Yes

Are sufficient details of methods and analysis provided to allow replication by others?

Yes

Reviewer Expertise:

Strategic management, business administration, and economist,

I confirm that I have read this submission and believe that I have an appropriate level of expertise to confirm that it is of an acceptable scientific standard.

# Reviewer report: Reviewer response for version 2

**DOI:** 10.5256/f1000research.161194.r241863

**Published:** 2024-02-27

**Authors:** Daniel Penido L. Amorim

**Affiliations:** 1 Universidade Federal de Minas Gerais, Belo Horizonte, State of Minas Gerais, Brazil

The authors did not make the suggested changes.

Is the work clearly and accurately presented and does it cite the current literature?

Partly

If applicable, is the statistical analysis and its interpretation appropriate?

Partly

Are all the source data underlying the results available to ensure full reproducibility?

Partly

Is the study design appropriate and is the work technically sound?

Partly

Are the conclusions drawn adequately supported by the results?

Partly

Are sufficient details of methods and analysis provided to allow replication by others?

Partly

Reviewer Expertise:

Financial Economics; Econometrics

I confirm that I have read this submission and believe that I have an appropriate level of expertise to state that I do not consider it to be of an acceptable scientific standard, for reasons outlined above.

# Reviewer report: Reviewer response for version 1

**DOI:** 10.5256/f1000research.134892.r185960

**Published:** 2023-08-15

**Authors:** Sanjib Biswas

**Affiliations:** 1 Calcutta Business School, Kolkata, India

The paper addresses a relevant issue which is however not very new. I find the following concerns

1. The paper suffers from flow and connectivity issues
2. Why will a researcher be interested to read this article? What is the value addition?
3. Why have the authors undertaken this work?
4. Literature review is outdated. It is required to study the papers published in 2020-2023.
5. The selection of methodology needs justification
6. The results need to be aligned with the key research questions and limitations need to be elaborated

Is the work clearly and accurately presented and does it cite the current literature?

Partly

If applicable, is the statistical analysis and its interpretation appropriate?

Partly

Are all the source data underlying the results available to ensure full reproducibility?

Yes

Is the study design appropriate and is the work technically sound?

Partly

Are the conclusions drawn adequately supported by the results?

Partly

Are sufficient details of methods and analysis provided to allow replication by others?

Partly

Reviewer Expertise:

Multicriteria Decision Making, Multivariate analysis, Logistics & Supply Chain Management, Sustainability

I confirm that I have read this submission and believe that I have an appropriate level of expertise to confirm that it is of an acceptable scientific standard, however I have significant reservations, as outlined above.

# Response

**Published:** 2024-01-04

**Authors:** Esin Benhür aktürk

**Affiliations:** istanbul aydin university, Turkey

First of all, we’d like to thank the respected professor reviewer for invaluable comments. We consider all these comments as crucial to improve our study. You can kindly find responses to your comment as follows:

**1. **The paper suffers from flow and connectivity issues

 We improved this issue. Thank you very much.

**2. **Why will a researcher be interested to read this article? What is the value addition?

 The study aims to assist investors in the stock market with market timing. By examining the relationship between macroeconomic indicators and the hot and cold issue market, it is intended to provide higher returns to investors. Investors can use macroeconomic indicators as a signal to gain insight into the trend of initial public offerings (IPOs) and, accordingly, time their investments to achieve higher short-term returns.

**3. **Why have the authors undertaken this work?

 The reason for our study is the relatively limited presence of research in the literature examining the relationship between stock returns and IPO trends. Through this study, we aim to contribute to the literature. We believe that the findings of this analysis, particularly focusing on the developed G-7 countries' capital markets, could serve as guiding insights for researchers delving into this subject.

**4. **Literature review is outdated. It is required to study the papers published in 2020-2023.

 As mentioned earlier, there exists a significant subject area in the literature on this matter. Consequently, the number of studies available is limited. Furthermore, due to its publication in 2021, this study naturally does not encompass research from the years 2022 and 2023. Nevertheless, three relevant studies published between 2021 and 2023 have been included in the literature.

**5. **The selection of methodology needs justification 6.The results need to be aligned with the key research questions and limitations need to be elaborated.

 In panel data analysis, the dataset encapsulates information from different cross-sections (e.g., various firms or countries) and across different time dimensions (e.g., different time periods). The Discroll-Kraay regression analysis is a method utilized for analyzing data within a panel data structure. This method specifically provides robust predictions against common issues such as autocorrelation and heteroscedasticity present in panel datasets.

 The Discroll-Kraay method is recommended to address concerns like autocorrelation and heteroscedasticity in panel data analysis. Autocorrelation denotes the situation where relationships between observations remain similar over time, whereas heteroscedasticity indicates varying variance among observations. By addressing such scenarios, this method ensures more reliable and robust regression outcomes.

 Moreover, the Discroll-Kraay method is widely recognized in the literature and is acknowledged for its reliability and effectiveness in panel data analysis. Hence, this method has been chosen for panel data analysis and forms part of the reasons behind its selection in this study.

# Reviewer report: Reviewer response for version 1

**DOI:** 10.5256/f1000research.134892.r182871

**Published:** 2023-07-14

**Authors:** Daniel Penido L. Amorim

**Affiliations:** 1 Universidade Federal de Minas Gerais, Belo Horizonte, State of Minas Gerais, Brazil

The article "Macro-economic determinants of the IPO waves: An assessment of G-7 countries" aimed to analyze which macroeconomic factors affect the number of IPOs in the G7 countries. The authors estimated a static model using a conventional panel data econometric method (random effects model). The results suggest that stock market returns influence the number of IPOs.

 The results are not new. Many articles on the topic have also found positive effects of stock market returns (or prices) on IPOs. However, the study contributes to the literature because it considers the countries that compose the G7. Adding this contribution and others in the introduction would be relevant to improving the article. Except for this, the introduction is good.

 Regarding the literature review, some citations are missing in the first paragraph of this section. The way the authors wrote the paragraphs, dedicating each to a single study, is not convenient. The authors should discuss the articles together, according to some questions they have in common. Furthermore, the authors need to add some articles on IPOs that are more recent. I list these articles at the end of this referee report.

 It is necessary to add at least the variables of interest rate and economic policy uncertainty (Backer et al., 2016) to the analysis. In several previous studies, the authors considered the interest rate in the models. Effects of economic policy uncertainty have appeared in recent studies such as Thanh (2020) and Amorim et al. (2021).

 The authors stated that the data used had a normal distribution, but this is not what the Jarque-Bera test suggests for some variables in the table of summary statistics. Moreover, the authors can remove the less common summary statistics from this table. IPO number data from Statista does not appear to be openly available, even after a basic registration.

 The more critical problems in the article arise from the econometric methods employed. The Hausman test is an asymptotic method. Therefore, it is not convenient to choose between fixed effects and random effects models based on it when the panel has only seven countries.

 Still on the models, we know that the process of deciding to go public (IPO) is dynamic. Agents can make these decisions with information from past periods. Therefore, it is common that the empirical models include lags. Moreover, stock prices affect IPOs because of the choice to go public in periods that favor IPO proceeds. However, IPOs also affect stock prices because these events cause stock market fluctuations. Thus, endogeneity is probable. Finally, there is also cross-sectional dependence. This requires the use of more advanced econometric methods than conventional panel data estimators with robust standard errors.

 I recommend that the authors estimate a dynamic model for long panel data, specifically the cross-sectionally augmented ARDL (CS-ARDL) approach (Chudik & Pesaran, 2015). The lags included in this model will overcome endogeneity and autocorrelation. The author can apply this method with variables I(0) (stationary) and I(1) (with a unit root). In addition, the method deals with cross-sectional dependence. In this approach, it has been common to use some tests jointly (two of them even the authors have used), which the authors can apply using the Stata software:

1. Pesaran's (2007) CIPS unit root test [command xtcips];
2. Pesaran’s (2021) cross-sectional dependence CD test [command xtcd];
3. Westerlund's (2007) cointegration test [command xtwest];
4. Chudik and Pesaran's (2015) CS-ARDL estimator [command xtdcce2]

These methods appear in studies such as Herzer (2019), Vo et al. (2022), and Uddin et al. (2023). Other dynamic models (for example, DOLS and FMOLS estimators) can be useful as robustness tests.

 Finally, the article needs to have the text revised. The authors need to improve their writing. Firstly, they need to use accurate vocabulary. For example, it is better to write "evidence indicates" or "evidence suggests" when referring to the results (no single study has a result "determinant"). There are well-known concepts that the authors miswrite, such as "resistant standard estimator" (I believe they meant "robust standard error" or "robust estimator") and "macro-economics" (do not use a hyphen). Furthermore, authors should avoid adopting passive voice in English and writing very long sentences. Some tools, such as Grammarly and Quillbot, contribute to the improvement of texts written by non-native English speakers.

 I hope the authors will continue to seek publication of the article.

**References**

 Ameer, R. (2012). Macroeconomic factors and initial public offerings (IPOs) in Malaysia. *Asian Academy of Management Journal of Accounting and Finance, 8*(1), 41–67. https://ejournal.usm.my/aamjaf/article/view/aamjaf_vol8-no1-2012_3

 Amorim, D. P. L., Camargos, M. A., & Ferreira, C. M. B. L. (2021). Macroeconomic Factors and Initial Public Offerings in Brazil. *Global Business Review*. https://doi.org/10.1177/09721509211003696

 Baker, S. R., Bloom, N., & Davis, S. J. (2016). Measuring economic policy uncertainty. *The Quarterly Journal of Economics, 131*(4), 1593–1636. https://doi.org/10.1093/qje/qjw024

 Chudik, A., & Pesaran, M. H. (2015). Common correlated effects estimation of heterogeneous dynamic panel data models with weakly exogenous regressors. *Journal of Econometrics, 188*(2), 393–420. https://doi.org/10.1016/j.jeconom.2015.03.007

 Deloof, M., de Jong, A., & Legierse, W. (2022). Going public: evidence from stock and bond IPOs in Belgium, 1839–1935. *Cliometrica.* https://doi.org/10.1007/s11698-022-00258-5

 Herzer, D. (2018). The long-run effect of aid on Health: Evidence from panel cointegration analysis. *Applied Economics, 51*(12), 1319–1338. https://doi.org/10.1080/00036846.2018.1527449

 Mehmood, W., Mohd-Rashid, R., & Ahmad, A. H. (2020). The variability of IPO issuance: evidence from Pakistan stock exchange. *Global Business Review.* https://doi.org/10.1177/0972150920929198

 Mehmood, W., Mohd-Rashid, R., Abdullah, Y. et al. (2023). Inclusive mapping of initial public offerings: a bibliometric and literature review study. *Quality & Quantity, 57*, 655–700. https://doi.org/10.1007/s11135-022-01387-9

 Mehmood, W., Mohd-Rashid, R., Ong, C.Z., & Abbas, Y.A. (2021). Factors driving IPO variability: evidence from Pakistan stock exchange. *Journal of Economics, Finance and Administrative Science, 26*(52), 300-316. https://doi.org/10.1108/JEFAS-04-2021-0036

 Pesaran, M. H. (2007). A simple panel unit root test in the presence of cross-section dependence. *Journal of Applied Econometrics, 22*(2), 265–312. https://doi.org/10.1002/jae.951

 Pesaran, M. H. (2020). General diagnostic tests for cross-sectional dependence in panels. *Empirical Economics, 60*(1), 13–50. https://doi.org/10.1007/s00181-020-01875-7

 Uddin, I., Ahmad, M., Ismailov, D., Balbaa, M. E., Akhmedov, A., Khasanov, S., & Haq, M. U. (2023). Enhancing institutional quality to boost economic development in developing nations: New insights from CS-ARDL approach. *Research in Globalization, 7*, 100137. https://doi.org/10.1016/j.resglo.2023.100137

 Vo, D. H., Ho, C. M., Le, Q. T., & Vo, A. T. (2022). Revisiting the energy-growth-environment nexus in the OECD countries: An application of the CS-ARDL approach. *Energy, Sustainability and Society, 12*(1). https://doi.org/10.1186/s13705-022-00375-z

 Westerlund, J. (2007). Testing for error correction in panel data. *Oxford Bulletin of Economics and Statistics, 69*(6), 709–748. https://doi.org/10.1111/j.1468-0084.2007.00477.x

Is the work clearly and accurately presented and does it cite the current literature?

Partly

If applicable, is the statistical analysis and its interpretation appropriate?

Partly

Are all the source data underlying the results available to ensure full reproducibility?

Partly

Is the study design appropriate and is the work technically sound?

Partly

Are the conclusions drawn adequately supported by the results?

Partly

Are sufficient details of methods and analysis provided to allow replication by others?

Partly

Reviewer Expertise:

Financial Economics; Econometrics

I confirm that I have read this submission and believe that I have an appropriate level of expertise to state that I do not consider it to be of an acceptable scientific standard, for reasons outlined above.

## References

[1] : Macroeconomic factors and initial public offerings (IPOs) in Malaysia. *Asian Academy of Management Journal of Accounting and Finance* .2012;1(8) :41-67 Reference source

[2] : Macroeconomic Factors and Initial Public Offerings in Brazil. *Global Business Review* .2021; 10.1177/09721509211003696 10.1177/09721509211003696

[3] : Measuring Economic Policy Uncertainty*. *The Quarterly Journal of Economics* .2016;131(4) : 10.1093/qje/qjw024 1593-1636 10.1093/qje/qjw024

[4] : Common correlated effects estimation of heterogeneous dynamic panel data models with weakly exogenous regressors. *Journal of Econometrics* .2015;188(2) : 10.1016/j.jeconom.2015.03.007 393-420 10.1016/j.jeconom.2015.03.007

[5] : Going public: evidence from stock and bond IPOs in Belgium, 1839–1935. *Cliometrica* .2022; 10.1007/s11698-022-00258-5 10.1007/s11698-022-00258-5

[6] : The long-run effect of aid on health: evidence from panel cointegration analysis. *Applied Economics* .2019;51(12) : 10.1080/00036846.2018.1527449 1319-1338 10.1080/00036846.2018.1527449

[7] : The Variability of IPO Issuance: Evidence from Pakistan Stock Exchange. *Global Business Review* .2020; 10.1177/0972150920929198 10.1177/0972150920929198

[8] : Inclusive mapping of initial public offerings: a bibliometric and literature review study. *Quality & Quantity* .2023;57(1) : 10.1007/s11135-022-01387-9 655-700 10.1007/s11135-022-01387-9

[9] : Factors driving IPO variability: evidence from Pakistan stock exchange. *Journal of Economics, Finance and Administrative Science* .2021;26(52) : 10.1108/JEFAS-04-2021-0036 300-316 10.1108/JEFAS-04-2021-0036

[10] : A simple panel unit root test in the presence of cross-section dependence. *Journal of Applied Econometrics* .2007;22(2) : 10.1002/jae.951 265-312 10.1002/jae.951

[11] : General diagnostic tests for cross-sectional dependence in panels. *Empirical Economics* .2021;60(1) : 10.1007/s00181-020-01875-7 13-50 10.1007/s00181-020-01875-7

[12] : Enhancing institutional quality to boost economic development in developing nations: New insights from CS-ARDL approach. *Research in Globalization* .2023;7: 10.1016/j.resglo.2023.100137 10.1016/j.resglo.2023.100137

[13] : Revisiting the energy-growth-environment nexus in the OECD countries: An application of the CS-ARDL approach. *Energy, Sustainability and Society* .2022;12(1) : 10.1186/s13705-022-00375-z 10.1186/s13705-022-00375-z

[14] : Testing for Error Correction in Panel Data. *Oxford Bulletin of Economics and Statistics* .2007;69(6) : 10.1111/j.1468-0084.2007.00477.x 709-748 10.1111/j.1468-0084.2007.00477.x

# Reviewer report: Reviewer response for version 1

**DOI:** 10.5256/f1000research.134892.r177271

**Published:** 2023-06-28

**Authors:** Prof.Dr.Cemal Zehir

**Affiliations:** 1 Yıldız Technical University, Istanbul, Turkey

In this peer-reviewed article, it has been examined whether the macroeconomic factors that are thought to be effective on the public offering cycle, and especially economic growth, inflation and stock market returns, are determinants of the number of public offerings. From this point of view, it will contribute to the literature.

 The analysis employs a random effect model and applies the Driscoll-Kraay resistant standard estimator to account for deviations.

 The findings reveal that stock market returns have a positive and significant effect on the volume of public offerings. This suggests that companies tend to time their IPOs during periods when stock market returns are relatively high.

 This research provides insights into the factors influencing companies' decision-making processes regarding IPO timing, specifically emphasizing the role of macroeconomic indicators. By understanding the relationship between stock market performance and IPO frequency, policymakers and investors can gain valuable insights for strategic decision-making.

 The analysis has been applied to the G-7 countries representing the developed world economies for the period 1999-2020. The countries analyzed and the fact that it covers a period of 20 years will be useful information for the readers.

 Research results are also a guide for practitioners. This article is suitable for publication.

Is the work clearly and accurately presented and does it cite the current literature?

Yes

If applicable, is the statistical analysis and its interpretation appropriate?

Yes

Are all the source data underlying the results available to ensure full reproducibility?

Yes

Is the study design appropriate and is the work technically sound?

Yes

Are the conclusions drawn adequately supported by the results?

Yes

Are sufficient details of methods and analysis provided to allow replication by others?

Yes

Reviewer Expertise:

Strategic management, business administration, and economist,

I confirm that I have read this submission and believe that I have an appropriate level of expertise to confirm that it is of an acceptable scientific standard.
